# Comparison of Transcriptome Responses between *Sogatella furcifera* Females That Acquired Southern Rice Black-Streaked Dwarf Virus and Not

**DOI:** 10.3390/insects13020182

**Published:** 2022-02-09

**Authors:** Dandan Liu, Zhengxi Li, Maolin Hou

**Affiliations:** 1 State Key Laboratory for Biology of Plant Diseases and Insect Pests, Institute of Plant Protection, Chinese Academy of Agricultural Sciences, Beijing 100193, China; liudandan2016@yeah.net; 2 College of Plant Protection, China Agricultural University, Beijing 100193, China; zxli@cau.edu.cn

**Keywords:** *Sogatella furcifera*, southern rice black-streaked dwarf virus, transcriptomic analysis, caspase 1, female-specific genes

## Abstract

**Simple Summary:**

The southern rice black-streaked dwarf virus (SRBSDV) is transmitted horizontally by the planthopper, *Sogatella furcifera*. During feeding on virus-infected plants, *S. furcifera* may acquire or fail to acquire SRBSDV. In this study, the responses were compared among the *S. furcifera* successfully acquiring the virus, those failing to acquire the virus, and those not exposed to SRBSDV (the control). A total of 1043 and 2932 differentially expressed genes (DEGs) were obtained in *S. furcifera* females that acquired SRBSDV and that failed to, in comparison with the control, respectively. Functionally, these DEGs are primarily involved in diverse signaling pathways related to primary metabolism and innate immunity, such as apoptosis. Additional bioassays confirmed the activation of apoptosis in *S. furcifera* by SRBSDV exposure. Interestingly, we also found that six female-specific genes were also upregulated in *S. furcifera* females exposed to SRBSDV. Our results further the understanding of the interactions between the vector *S. furcifera* females and SRBSDV at the molecular level.

**Abstract:**

The southern rice black-streaked dwarf virus (SRBSDV) is transmitted horizontally by *Sogatella furcifera* in a persistent, propagative manner. Exposure of *S. furcifera* females to SRBSDV-infected rice plants may trigger transcriptomic changes in the insects, the transcriptomes of females that acquired SRBSDV and those that failed to, as well as females fed on healthy rice plants as control, were sequenced and compared. Nine transcriptomic libraries were constructed, from which a total of 53,084 genes were assembled. Among the genes, 1043 and 2932 were differentially expressed genes (DEGs) in *S. furcifera* females that acquired SRBSDV and that failed to, in comparison with the control, respectively. Functional enrichment analysis showed that DEGs identified in *S. furcifera* females exposed to SRBSDV are primarily involved in diverse signaling pathways related to primary metabolism and innate immunity. The DEGs in the *S. furcifera* females that failed to acquire the virus significantly outnumbered that in the insects that acquired the virus, and the virus exposure activated the humoral and cellular immune responses of the vectors, especially the apoptosis. The key gene in apoptosis encoding caspase 1 was upregulated by SRBSDV exposure, especially in *S. furcifera* females that failed to acquire the virus. Analysis of caspase 1 activity validated that SRBSDV exposure induced caspase 1 accumulation. Surprisingly, the expression of six female-specific genes was also upregulated by SRBSDV exposure, which was confirmed by RT-qPCR analysis. This study provides evidence to explain the differential virus acquisition at the transcriptome level.

## 1. Introduction

The white-backed planthopper, *Sogatella furcifera* Horváth (Hemiptera: Delphacidae), is a dimorphic and migratory insect pest. It causes tremendous loss in rice production by sucking phloem sap from leaf sheath and transmitting the rice virus [1,2,3]. Southern rice black-streaked dwarf virus (SRBSDV) is a devastating virus transmitted exclusively by *S. furcifera* in a persistent and propagative manner [3,4,5,6,7].

The interactions between host plant, vector, and virus are complex. In the case of SRBSDV, virus acquisition is higher at 32 °C and 27 °C than at 22 °C, from tillering stage plants than from three-leaf stage and booting stage plants, and by third instars than by fifth instars [8]. The infection of SRBSDV induces physiological and/or behavioral changes in *S. furcifera*. SRBSDV-infected *S. furcifera* nymphs develop slower [9,10], and viruliferous females are less fecund [11], which may influence the transmission of SRBSDV. Viruliferous *S. furcifera* species spend more time in salivation and phloem sap ingestion on healthy rice plants than nonviruliferous *S. furcifera* [9,12] and infected plants show an arrestant effect on nonviruliferous *S. furcifera* for phloem sap ingestion [10]. The acquisition of plant viruses depends on the vector’s orientation to and feeding on virus-infected plants. However, in many virus–vector–plant systems, vector feeding does not always result in virus acquisition [13]. It was determined that the *S. furcifera* that are quicker to reach phloem and spend more time in phloem sap ingestion stand a high chance of virus acquisition [10].

Recently, transcriptome sequencing has been widely applied in the study of the interactions between insect vectors and plant viruses [14]. In the *Bemisia tabaci*—tomato yellow leaf curl China virus (TYLCCNV) system, 1606 differentially expressed genes (DEGs) were identified in viruliferous whiteflies, which provides a road map for an investigation into the complex interactions in the system and partially explains the negative effects of the virus on the longevity and fecundity of *B. tabaci* [15]. Comparative genomics analysis advanced a repertoire of candidate genes that might be involved in the interactions between *F. occidentalis* and TSWV [16]. Xu et al., (2012) constructed transcriptomes of mixed genders of viruliferous and nonviruliferous *S. furcifera* and found that SRBSDV infection modulated the immune regulatory systems to defend against virus infection, which was echoed in the immune response of *S. furcifera* males [17,18]. However, it is not clear if the transcriptome responses differ between *S. furcifera* females that have successfully acquired SRBSDV and those that have failed to.

Considering the specificity of a single sample population and the influence of SRBSDV on *S. furcifera* female physiological processes, we explored the response of *S. furcifera* females exposed to SRBSDV at a whole-transcriptomic level in this study. RNA sequencing technology was used to obtain the transcriptomes of *S. furcifera* females that acquired SRBSDV, those that failed to acquire SRBSDV, and those not exposed to SRBSDV-infected rice plants. Mass gene expression profiling was analyzed using bioinformatics, and the bioassays were used to detect caspase 1 in the processes of apoptosis. The present results provide gene information that may function in terms of facilitating virus acquisition by replication in the interaction of *S. furcifera* females and SRBSDV.

## 2. Materials and Methods

### 2.1. Insects and Plants

A nonviruliferous *S. furcifera* population was maintained on potted rice plants (var. Taichung Native 1, TN1) in a greenhouse (30 ± 5 °C, 15L:9D). A stock culture of SRBSDV-infected rice plants was established from SRBSDV-infected rice plants originating from the paddy fields in Xing’an (25°36′18″ N, 110°42′16″ E), Guangxi Zhuang Autonomous Region. The SRBSDV infection status of the rice plants was detected individually by one-step RT–PCR based on the 10th segments of SRBSDV genomes [19].

### 2.2. RNA Isolation, cDNA Library Preparation, and Transcriptome Sequencing

Newly emerged *S. furcifera* females were confined with SRBSDV-infected rice plants (10–15 insects per plant) for 2 days and then transferred to healthy rice plants. After 7 days, the insects were individually extracted for RNA (20 μL) using Total RNA Extraction Kit (Solarbio Science and Technology) following the manufacturer’s procedure. SRBSDV infection status of the insects was individually confirmed by RT–PCR using 2 μL of the RNA extract. According to SRBSDV infection status, the SRBSDV-exposed insects were classified into two groups, *S. furcifera* females that acquired SRBSDV (Sf–V) and those that failed to (Sf–NV). The remaining RNA extract (18 μL) was each diluted to 300 ng/μL with RNase-free water, and then, five RNA samples from the same *S. furcifera* group (either Sf–V or Sf–NV) were randomly pooled together for cDNA library construction and transcriptome sequencing. *Sogatella furcifera* females fed with healthy rice plants were used as the control (CK). For each group, three biological repetitions were performed for cDNA library construction and transcriptome sequencing.

The quantity and purity of RNA samples were analyzed using Bioanalyzer 2100 and RNA 1000 Nano LabChip Kit (Agilent, CA, USA) with RIN number > 7.0. Poly(A) RNA was purified from total RNA (5 μg) using poly-T oligo-attached magnetic beads through two rounds of purification. Following purification, the mRNA was fragmented into small pieces using divalent cations under elevated temperatures. Then, the cleaved RNA fragments were reverse-transcribed to create the final cDNA library in accordance with the protocol of the mRNASeq sample preparation kit (Illumina, San Diego, CA, USA). The average insert size for the paired-end libraries was 300 bp (±50 bp). Then, the paired-end sequencing was performed on an Illumina Hiseq4000 following the vendor’s recommended protocol (LC Sciences, HangZhou city, China).

### 2.3. De Novo Assembly, Gene Annotation, and Functional Classification

To obtain clean high-quality data, the reads that contained adaptor contamination, low-quality bases, and undetermined bases were removed using Cutadapt and Perl scripts in house [20]; then, sequence quality was verified using FastQC (http://www.bioinformatics.babraham.ac.uk/projects/fastqc/, accessed on 17 September 2021) including the Q20, Q30, and GC content of the clean data. All downstream analyses were based on clean, high-quality data. De novo assembly of the transcriptome was performed with Trinity 2.4.0 [21]. Trinity groups transcripts into clusters based on shared sequence content. The longest transcript in the cluster was chosen as the gene sequence (denoted as “Gene”).

For gene annotation and functional classification, all assembled genes were aligned against the nonredundant (Nr) protein database (http://www.ncbi.nlm.nih.gov/, accessed on 17 September 2021), Gene Ontology (GO) (http://www.geneontology.org, accessed on 17 September 2021), SwissProt (http://www.expasy.ch/sprot/, accessed on 17 September 2021), Kyoto Encyclopedia of Genes and Genomes (KEGG) (http://www.genome.jp/kegg/, accessed on 17 September 2021), and evolutionary genealogy of genes: Nonsupervised Orthologous Groups (eggNOG) (http://eggnogdb.embl.de/, accessed on 17 September 2021) databases using DIAMOND with a threshold of E-value < 0.00001 [22].

### 2.4. Analysis for Differentially Expressed Genes

Salmon was used to perform expression levels of genes by calculating TPM [23,24]. The differentially expressed genes (DEGs) were selected with |log2 (fold change)| > 1 and with statistical significance (*p* < 0.05) by R package edgeR [25]. GO and KEGG enrichment analyses were conducted using Scripts in house 1.3 and R package ggplot2.

### 2.5. Expression of Caspase 1 and Female-Specific Genes

According to the results of DEGs analysis, the apoptosis-related genes and 6 genes in the top 10 upregulated female-specific genes were upregulated by SRBSDV exposure; thus, we analyzed the expression of the key gene in apoptosis, *S. furcifera* caspase 1 (*Sfcas1*), and these female-specific genes. Real-time quantitative PCR (RT–qPCR) analysis was used to detect the expression levels of *Sfcas1* and female-specific genes in Sf–V, Sf–NV, and CK females using the ABI 7500 Real-Time PCR system (Applied Biosystems, Foster City, CA, USA) and an SYBR Green Master Mix (Takara Biomedical Technology, Beijing, China). The RT–qPCR conditions were as follows: 95 °C for 30 s, 40 cycles of 95 °C for 5 s, and finally, 60 °C for 30 s, followed by an analysis of melting curve to test the purity of RT–qPCR. *Sogatella furcifera* housekeeping gene elongation factor 1-α (*EF1α*) was detected in parallel as an internal control [26]. The primers of Sfcas1, female-specific genes, and *SfEF1α* were designed using Primer 5. The efficiency of the primers was validated before gene expression analysis. All the specific primers used in RT–qPCR are listed in Table S1.

### 2.6. Caspase 1 Activity

Owing to the undetectability of the activity of caspase 1 from individual *S. furcifera* female, 20 females exposed to virus-infected rice plants for 2 d were used as one sample for detection of the activity of caspase 1. Briefly, the 20 females were ground in cell lysis buffer (Beyotime, Shanghai, China), and the total protein concentrations were quantified and normalized to 1 mg total proteins/mL samples; thereafter, the caspase 1 activity was assayed using a caspase-1 activity assay kit (Beyotime, Shanghai, China) according to the manufacturer’s instruction. The absorbance was measured at a wavelength of 405 nm by the multiplate reader Synergy 4 (BioTek, Winooski, VT, USA). The assay was biologically repeated three times for the CK females and the females exposed to virus-infected rice plants. According to our preliminary tests, more than 80% of *S. furcifera* females acquired SRBSDV when exposed to the virus-infected rice plants.

### 2.7. Data Analysis

The data of RT–qPCR was calculated using the comparative CT method (2^−△△Ct^) and normalized against *S. furcifera EF1α*. One-way analysis of variance (ANOVA) was used to detect the significance of the effects of exposure to SRBSDV on gene expression level. Where there was a significant effect, the Tukey test was used to separate the means when equal variance was assumed, or the Games–Howell test was used when equal variance was not assumed. The *t*-test was used to separate the means of caspase 1 activity levels of *S. furcifera* females exposed to SRBSDV-infected plants and those exposed to healthy plants. All data were analyzed using SPSS 23.0.

## 3. Results

### 3.1. Transcriptome Assembly and Annotation

**Figure 1:**
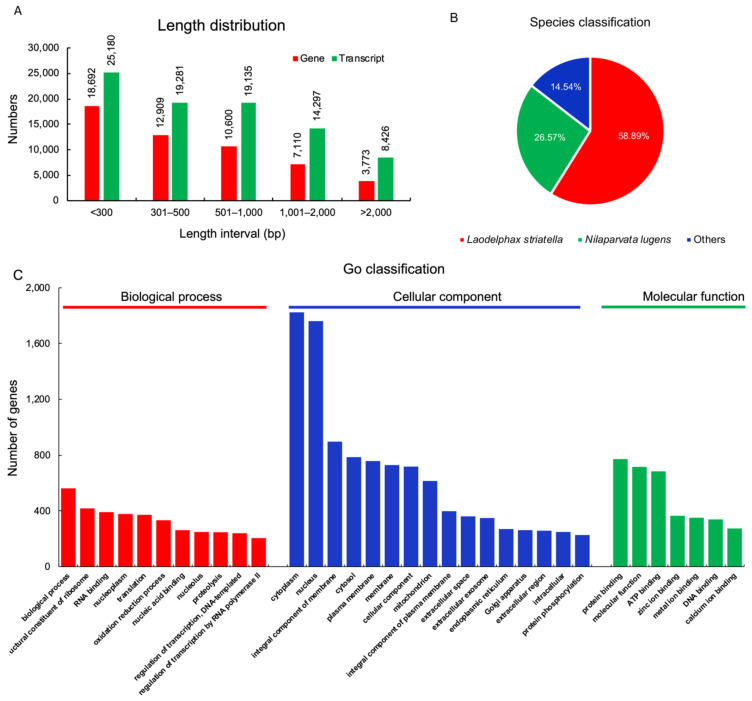
Summary of genes and transcripts in *S. furcifera* females: (**A**) length distribution of genes and transcripts; (**B**) sequence homology of *S. furcifera* genes to those of other insect species; (**C**) GO classification of genes.

The whole transcriptomes of SRBSDV-exposed *S. furcifera* females were constructed and archived at the NCBI Sequence Read Archive (SRA) under accession number PRJNA781429 (http://www.ncbi.nlm.nih.gov/sra, accessed on 18 December 2021). Using paired end-joining and gap-filling methods, all contigs were further assembled into scaffolds. In total, 453,978,036 valid bases were generated for nine libraries. The average Q30 value of these libraries was 95.06%, indicating the sequencing data were effective and reliable (Table 1). The 53,084 genes ranging from 201 bp to 11,242 bp with a median length of 395 bp were assembled (Table 2), of which 9650 (18.18%) and 17,102 (32.22%) genes had homologous sequences in the SwissProt and NR protein databases, respectively, and 10,802 (20.35%), 10,059 (18.95%), 13,198 (24.86%), and 10,543 (19.86%) genes were classified by GO, KEGG, eggNOG, and Protein family (Pfam) databases, respectively (Table 3). The length of 20.50% of the genes was longer than 1 kb (Figure 1A). When blasted against the NCBI NR protein database, the assembled genes were mostly (58.89%) homologous to the sequences of *Laodelphax striatella* and partially (26.57%) to *Nilaparvata lugens* sequences (Figure 1B). GO annotation revealed that biological process was the highest term among all terms in the biological process classification category. As regards cellular components, both cytoplasm and nucleus accounted for the most represented proportion. In molecular component, protein binding, molecular component, and ATP binding accounted for similar proportions (Figure 1C).

### 3.2. GO and KEGG Analyses of DEGs

**Figure 2:**
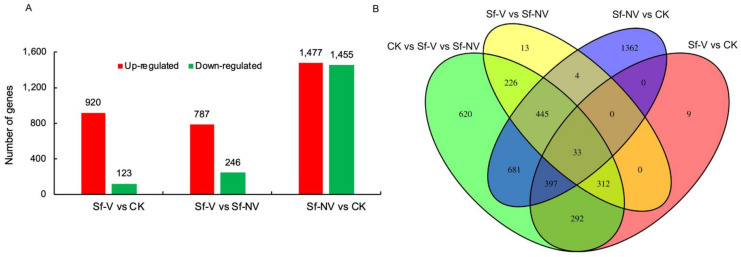
Differentially expressed genes (DEGs) between *S. furcifera* treatments: Sf–V, *S. furcifera* females acquired SRBSDV; Sf–NV, *S. furcifera* females failed to acquire SRBSDV; CK, *S. furcifera* females exposed to healthy rice plants: (**A**) up- and downregulated DEGs between treatments; (**B**) Venn diagram of shared DEGs between treatments.

The largest number of DEGs (2932) was detected in Sf–NV, compared with CK (Figure 2A), where 1477 genes were upregulated, 1455 genes were downregulated, and the |log2 Fold Change| ranged between 1 and 11.95. In comparison with CK, 1043 DEGs were obtained in Sf–V, where 920 genes were upregulated, 123 genes were downregulated, and the |log2 Fold Change| ranged between 1 and 12.16. In total, 430 genes were detected in both Sf–NV and Sf–V, compared with CK (Figure 2B). In comparison with Sf–NV, 1033 DEGs were detected in Sf–V, of which 787 genes were upregulated, 246 genes were downregulated (Figure 2A), and the |log2 Fold Change| between 1 and 12.48.

**Figure 3:**
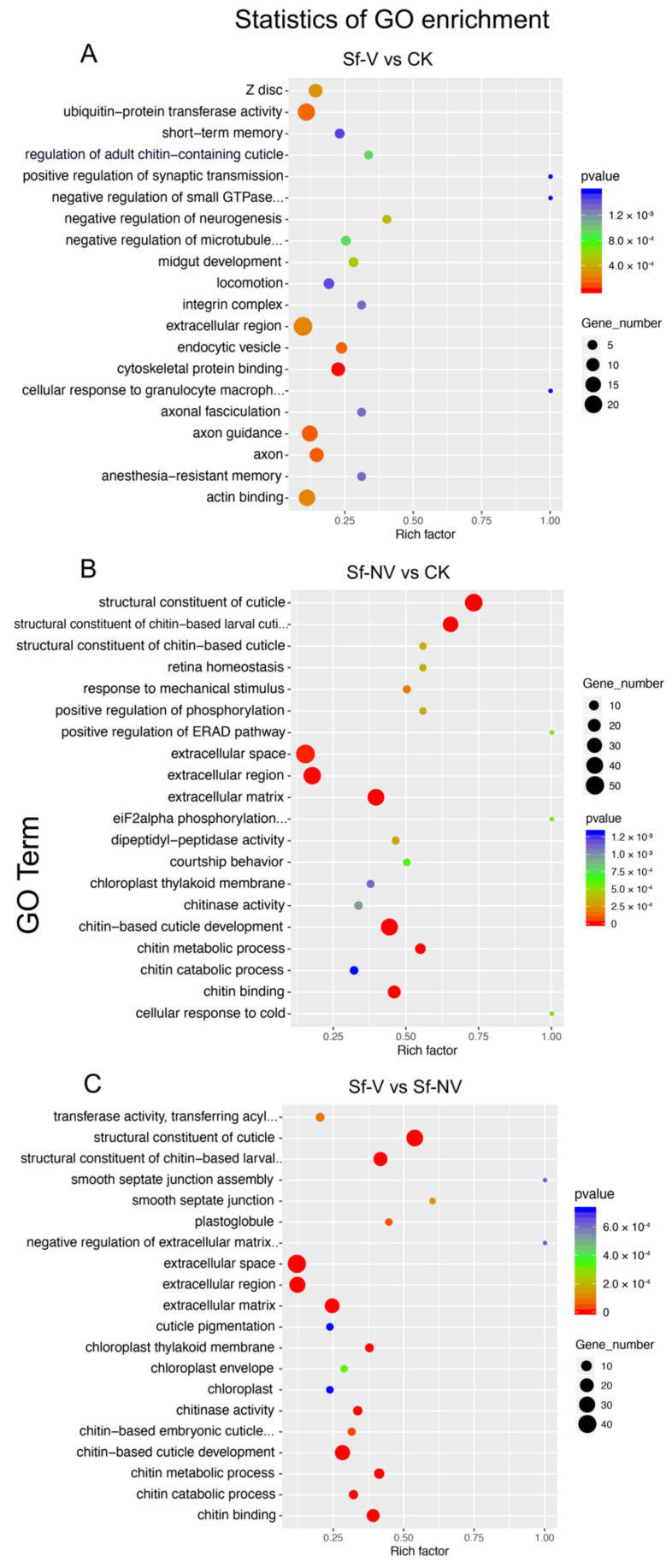
GO enrichment of differentially expressed genes. The enrichment factor was calculated by the numbers of DEGs in a term/the numbers of genes in the term, which reflects the degree of enrichment: Sf–V, *S. furcifera* females acquired SRBSDV; Sf–NV, *S. furcifera* females failed to acquire SRBSDV; CK, *S. furcifera* females exposed to healthy rice plants.

DEGs were further annotated by the GO function (http://www.geneontology.org/, accessed on 17 September 2021). In comparison with DEGs in CK, DEGs in Sf–V were significantly (*p* < 0.01) enriched in ubiquitin-protein transferase activity, extracellular region, axon guidance, and actin binding (Figure 3A). Compared with DEGs in CK, DEGs in Sf–NV were significantly (*p* < 0.01) enriched in the structural constituent of the cuticle, extracellular space, and extracellular region (Figure 3B). The most enriched functional process of DEGs in Sf–V, compared with Sf–NV, was in the structural constituent of the cuticle, extracellular space, extracellular region, and chitin-based cuticle development (Figure 3C).

**Figure 4:**
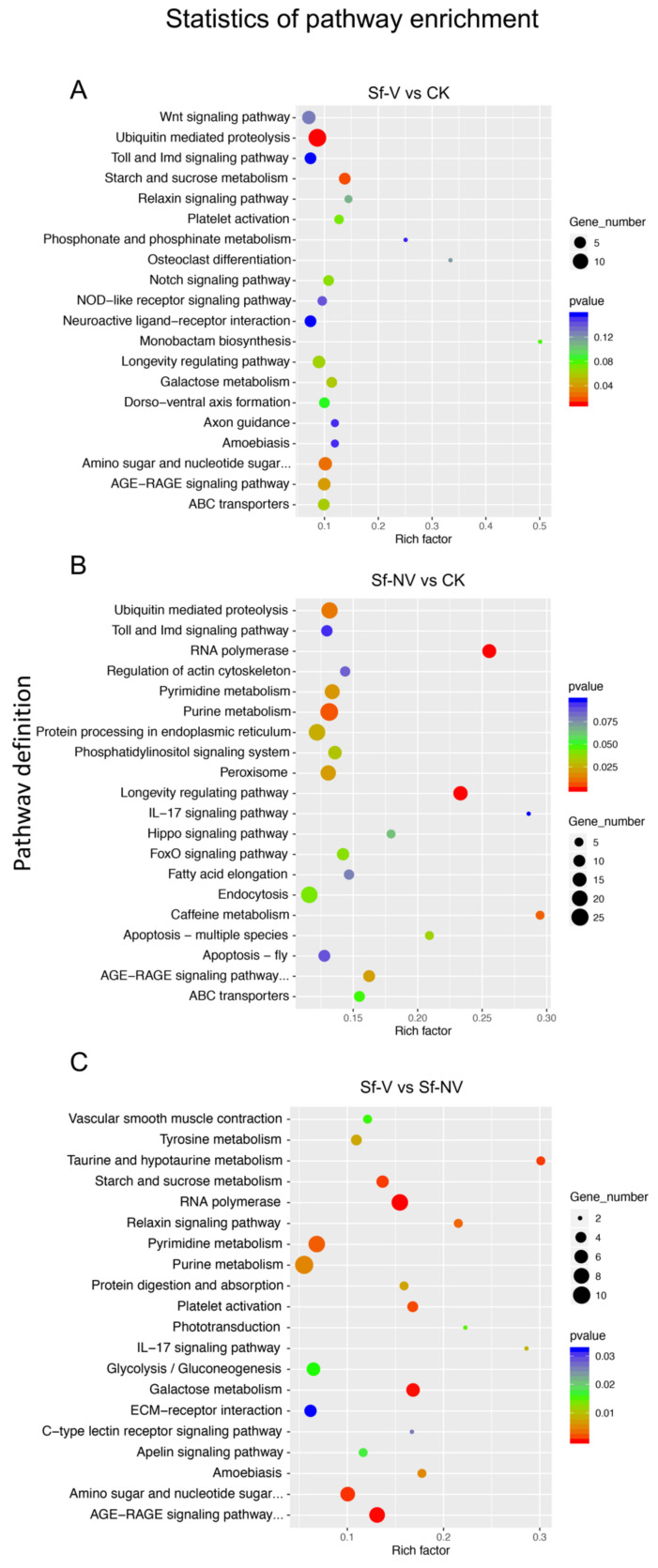
KEGG enrichment of differentially expressed genes. The enrichment factor was calculated by the numbers of DEGs in a pathway/the numbers of genes in the pathway, which reflects the degree of enrichment: Sf–V, *S. furcifera* females acquired SRBSDV; Sf–NV, *S. furcifera* females failed to acquire SRBSDV; CK, *S. furcifera* females exposed to healthy rice plants.

When analyzed by the KEGG pathway (http://www.genome.jp/kegg/, accessed on 17 September 2021), DEGs significantly enriched in Sf–V in comparison with CK were primarily associated with ubiquitin-mediated proteolysis and Wnt signaling pathway (Figure 4A). Overall, 14 DEGs enriched in the ubiquitin-mediated proteolysis were upregulated, and 1 gene was downregulated. Unlike DEGs in Sf–V, the most enriched pathways of DEGs in Sf–NV, compared with CK, were purine metabolism, endocytosis, and apoptosis (Figure 4B). Compared with DEGs in Sf–NV, DEGs in Sf–V were mostly enriched in RNA polymerase, purine metabolism, and pyrimidine metabolism (Figure 4C).

### 3.3. DEGs Associated with Apoptosis in S. furcifera Females

The DEGs associated with apoptosis were identified (Table 4). The expressions of 14 genes in 17 apoptosis-related genes were upregulated in Sf–NV *S. furcifera* females, and that of 6 genes were upregulated in Sf–V *S. furcifera* females. Four genes’ expression levels were significantly upregulated in both Sf–V and Sf–NV *S. furcifera* females, such as caspase 1 (a key gene in apoptosis).

**Figure 5:**
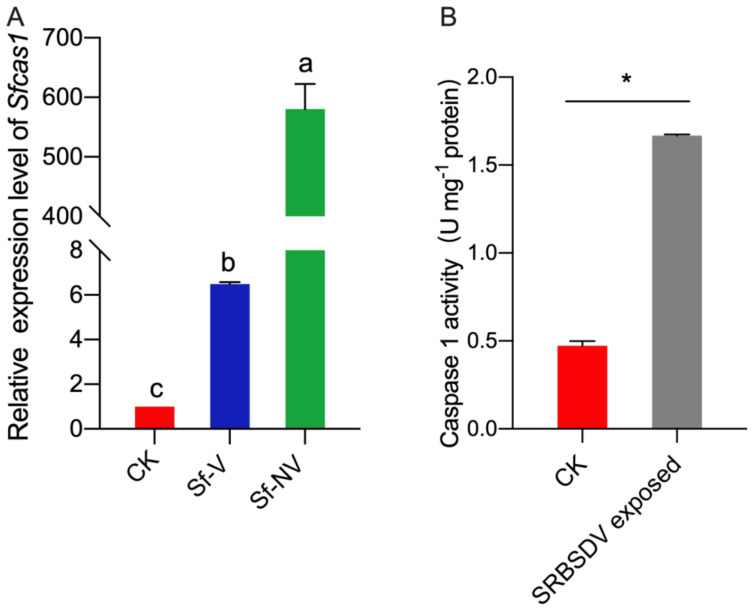
The Sfcas1 expression level and caspase 1 activity in *S. furcifera*: (**A**) expression of Sfcas1 in *S. furcifera* females acquired SRBSDV or not: Sf–V, *S. furcifera* females acquired SRBSDV; Sf–NV, *S. furcifera* females failed to acquire SRBSDV; CK, *S. furcifera* females exposed to healthy rice plants. Expression was normalized to the level of *SfEF1α* that serves as the internal standard. The relative mRNA level of Sfcas1 in *S. furcifera* fed on healthy rice plants (CK) was arbitrarily set to 1. Different letters over the bars (mean ± S.E.) indicate significant differences (Duncan test, *p* < 0.05); (**B**) caspase 1 activity in *S. furcifera* exposed to SRBSDV infected rice or not. “*” indicates significant difference (*t* test, *p* < 0.01).

The accumulation of key genes of apoptosis-encoding caspase 1 in *S. furcifera* (*Sfcas1*) was further confirmed. SRBSDV exposure significantly affected the expression of Sfcas1 (F = 562.5; df = 2,6; *p* < 0.01; Figure 5A). The expression level of Sfcas1 increased significantly in Sf–NV compared with that in CK or Sf–V *S. furcifera* females, and in Sf–V compared with that in CK *S. furcifera*. In *S. furcifera* exposed to SRBSDV-infected vs. to healthy rice plants, the activity of caspase 1 increased significantly (*t*-test, *p* < 0.01; Figure 5B).

### 3.4. Expression of Female-Specific Genes in S. furcifera

Among the top 10 female-specific genes identified in *S. furcifera* [27], 6 genes were found in the DEGs of *S. furcifera* females acquired SRBSDV or not in comparison with the control. Expressions of the six DEGs were significantly upregulated in Sf–NV females compared with those in CK females, while only three DEGs were more expressed in Sf–V females than in CK females (Table 5).

**Figure 6:**
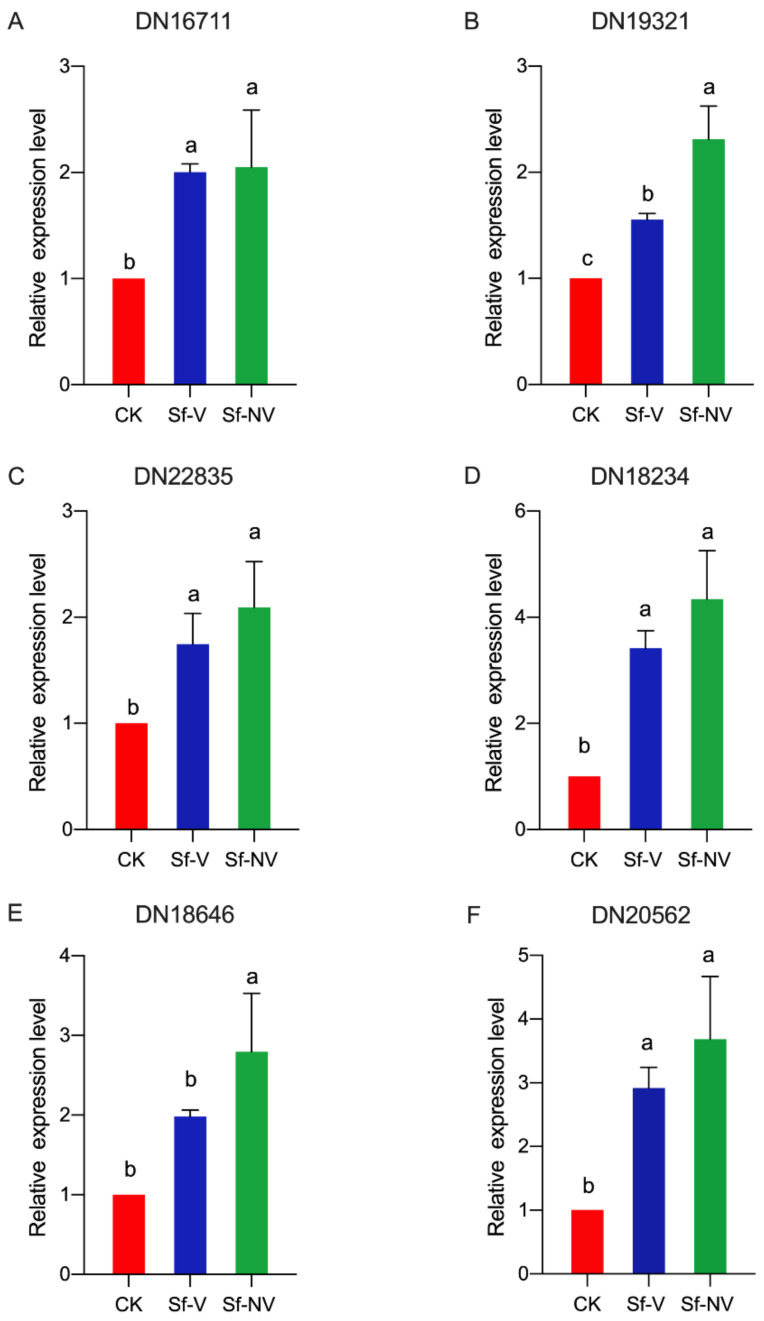
The expression levels of female-specific genes in *S. furcifera* acquired SRBSDV or not: Sf–V, *S. furcifera* females acquired SRBSDV; Sf–NV, *S. furcifera* females failed to acquire SRBSDV; CK, *S. furcifera* females exposed to healthy rice plants. Expression was normalized to the level of *SfEF1α* that serves as the internal standard. The relative mRNA levels of female-specific genes in CK were arbitrarily set to 1. Different letters over the bars (mean ± S.E.) indicate significant differences (Tukey test, *p* < 0.05).

Further detection of the six female-specific genes’ expression was conducted using RT–qPCR. The exposure of SRBSDV in *S. furcifera* females significantly affected the expression of gene DN16711 (F = 10.39; df = 2,6; *p* = 0.01; Figure 6A); DN19321 (F = 36.21, df = 2,6; *p* < 0.01; Figure 6B); DN22835 (F = 6.37, df = 2,6; *p* = 0.03; Figure 6C); DN18234 (F = 23.57; df = 2,6; *p* < 0.01; Figure 6D); DN18646 (F = 13.04; df = 2,6; *p* < 0.01; Figure 6E); DN20562 (F = 13.37; df = 2,6; *p* < 0.01; Figure 6F). In comparison with CK, the expression levels of female-specific genes increased significantly in Sf–V and Sf–NV *S. furcifera*. Especially, the expression levels of genes DN16711, DN19321, DN18646 in Sf–NV *S. furcifera* were significantly higher than Sf–V *S. furcifera*. These results were consistent with the transcriptome results.

## 4. Discussion

Plant viruses are primarily transmitted via insect vectors and cause tremendous economic loss in the world. There are complex interactions between viruses and vectors [28], which have been explored at the transcriptomic level in many vector–virus systems [14], such as *B. tabaci*–TYLCCNV [15], *F. occidentalis*–TSWV [16], *Schizaphis graminum*–barley yellow dwarf virus (BYDV) [29]. In the present study, the transcriptome of *S. furcifera* females was significantly modulated when females were exposed to SRBSDV-infected rice plants. In total, 1043 DEGs were up- or downregulated in *S. furcifera* females that acquired SRBSDV (Sf–V) (Figure 2), and 2932 DEGs in insects that failed to acquire virus (Sf–NV). Functional analysis of these DEGs showed that primary metabolism was modulated, which was consistent with the transcriptomic analysis of *S. furcifera* males or mixed populations infected with SRBSDV [17,18]. Remarkably, genes involved in the cellular and humoral immune response in *S. furcifera* females exposed to SRBSDV were upregulated in this research, while they were downregulated in *S. furcifera* males in previous research [18]. The pattern of immune responses observed in this study may be attributed to differences in insect genders (male vs. female), *S. furcifera* colony strains, and insect stages (second-instar nymphs vs. adult), as well as rice plants used for feeding. The discrepancies between these studies shed light on the need for additional investigations into *S. furcifera* at different stadium responses to SRBSDV.

Especially, the transcriptome of *S. furcifera* females that failed to acquire SRBSDV (Sf–NV) was established for the first time. The DEGs in Sf–NV significantly outnumbered those in Sf–V (Figure 2), indicating the stronger transcriptomic responses in Sf–NV than in Sf–V. In comparison, a previous study by Wang et al., (2016) used the male *S. furcifera* as a model and found 1444, 2237, and 2288 DEGs in the high virus titers group (HVT), medium virus titers group (MVT), and nonviruliferous group (NVF), respectively [18], which also implies more regulated genes in insects with different virus titers. Insects rely on their immune systems to fight against invading pathogens, such as viruses and bacteria [18]. Most of the DEGs detected in Sf–NV relative to CK are immune-related genes involved in endocytosis, apoptosis, peroxisome, and Toll and IMD signaling pathways (Figure 4), indicating that stronger immune responses are triggered in Sf–NV than in Sf–V. These results may partially explain the differential virus acquisition between *S. furcifera* females.

Apoptosis is a normal physiological process of programmed cell death (PCD) controlled by genes to maintain the stability and homeostasis of animals. Apoptosis plays a critical role in embryonic morphogenesis and development, immunity responses against pathogens, cellular damage, and aging [30,31]; moreover, it is a powerful effector response to virus infection and can reduce viral replication, infectivity, and spread within the insect host [32]. However, to date, apoptosis is largely unexplored in virus–insect interactions. Caspase is a family of cysteine proteases responsible for executing the apoptotic cascade, and caspase 1 is the critical factor in triggering apoptosis in animals [33,34,35,36]. In this study, SRBSDV exposure induced apoptosis in *S. furcifera* females (Table 4), as revealed by the heightened expression of *Sfcas1* and the increased activity of caspase 1 (Figure 5). Further studies are required for the function of *Sfcas1* during *S. furcifera* female transmitting SRBSDV, which may lead to the failure of SRBSDV acquisition in some *S. furcifera* females.

Interestingly, 6 genes in the top 10 upregulated female-specific genes [27] were even significantly upregulated in Sf–V and Sf–NV (Table 5). The RT–qPCR detection of these female-specific genes confirmed that SRBSDV exposure activated the expression of these genes, which were consistent with transcriptome results (Figure 5). The bioinformatics of these genes shows that these genes may be associated with DNA synthesis or the nervous system (Table 5), which may be related to their complicated physiological processes, such as fecundity and immune responses. Previous studies elucidated that the geminiviruses can activate DNA synthesis [37,38]. Additionally, using the GO enrichment and KEGG pathway mapping, we elucidated that *S. furcifera* exposed to SRBSDV upregulated genes involved in tissue regeneration (e.g., hippo signaling, Figure 4) and cell proliferation (e.g., notch signaling, Figure 4), which involves DNA synthesis. Whether these responses were caused by *S. furcifera* female repair after the viral disruption of tissue function remains to be explored.

## 5. Conclusions

In conclusion, the transcriptomic profiling of *S. furcifera* females reveals that the DEGs in *S. furcifera* females that failed to acquire the virus significantly outnumbered those in insects that acquired the virus. The DEGs identified in *S. furcifera* females exposed to SRBSDV are primarily involved in diverse signaling pathways related to primary metabolism, and the virus exposure activates the humoral and cellular immune responses of vectors, especially apoptosis. The bioassay of caspase 1 activity, RT–qPCR of the critical gene in apoptosis *Sfcas1*, and several female-specific genes confirm their roles in *S. furcifera* females in response to SRBSDV. The results of this study partially explained the differential virus acquisition at the transcriptome level.

## Figures and Tables

**Table 1:** Summary of RNA-seq metrics from the transcriptome sequencing data of *S. furcifera* females.

Treatment	Raw Reads	Valid Reads	Valid Bases	Q30 (%)	GC (%)
CK1	56,680,154	54,835,900	7.69 G	95.17	41.99
CK2	55,922,502	53,222,474	7.45 G	94.91	42.87
CK3	54,429,046	51,774,068	7.25 G	94.97	43.16
Sf_NV1	57,203,084	54,911,608	7.70 G	95.53	43.38
Sf_NV2	48,169,010	43,964,184	6.15 G	94.91	43.92
Sf_NV3	56,369,404	49,752,106	6.95 G	95.04	43.62
Sf_V1	42,071,366	37,432,246	5.24 G	94.7	44.16
Sf_V2	58,402,142	54,280,658	7.61 G	95.13	43.49
Sf_V3	54,980,040	53,804,792	7.54 G	95.18	41.26

Sf–V, *S. furcifera* females acquired SRBSDV; Sf–NV, *S. furcifera* females failed to acquire SRBSDV; CK, *S. furcifera* females exposed to healthy rice plants.

**Table 2:** Assembly of the transcripts and genes from transcriptome sequencing data of *S. furcifera* females.

Index	No. Transcripts	No. Genes
All	96,752	53,084
GC%	38.41	38.16
Min length (bp)	201	201
Median length (bp)	416	395
Max length (bp)	11,242	11,242
Total assembled Bases (bp)	69,880,720	37,703,068
N50 length (bp)	1164	1171

**Table 3:** Statistics of genes annotated by different databases in *S. furcifera* females.

Data Base	Number	Ratio (%)
All	53,084	100
GO	10,802	20.35
KEGG	10,059	18.95
Pfam	10,543	19.86
SwissProt	9650	18.18
eggNOG	13,198	24.86
NR	17,102	32.22

**Table 4:** Identification of DEGs associated with apoptosis in *S. furcifera* females.

Gene ID	Annotation	log2 FC		
		Sf–NV vs. CK	Sf–V vs. CK	Sf–V vs. Sf–NV
DN17359	actin 1	−1.33	4.33	5.69
DN19763	tubulin alpha-1A chain-like	1.71	/	/
DN22978	Ras GTPase-activating protein	1.18	1.67	/
DN24440	actin	−1.13	/	/
DN17338	dynamin-1-like protein	1.02	/	/
DN17887	Stress-activated protein kinase JNK	1.36	/	/
DN18498	cellular tumor antigen p53-like	1.64	/	/
DN19626	caspase 1	1.15	1.43	/
DN19706	serine-protein kinase	1.11	1.83	/
DN20330	cellular tumor antigen p53-like	1.16	/	/
DN21372	baculoviral IAP repeat-containing protein 6-like	1.11	/	/
DN22189	Poly [ADP-ribose] polymerase	1.09	1.65	/
DN22345	eukaryotic translation initiation factor 2-alpha kinase-like	1.29	/	/
DN17707	Apoptosis inhibitor IAP OS	1.14	/	/
DN25173	protein S100-A9	7.52	/	−7.44
DN17988	Cell division cycle and apoptosis regulator protein 1	1.39	/	/
DN20596	Dynamin-1-like protein	/	1.55	/

FC, fold change. |log2 FC| > 1 indicates significant difference. “/” indicates no significant differences (negative binomial (NB) distribution, *p* > 0.05): Sf–V, *S. furcifera* females acquired SRBSDV; Sf–NV, *S. furcifera* females failed to acquire SRBSDV; CK, *S. furcifera* females exposed to healthy rice plants.

**Table 5:** Identification of DEGs associated with female-specific genes in *S. furcifera* females.

Gene ID	Annotation	log_2_ FC		
		Sf–NV vs. CK	Sf–V vs. CK	Sf–V vs. Sf–NV
DN18646	Coiled-coil domain-containing protein 50	1.44	/	/
DN22835	Chromodomain-helicase-DNA-binding protein 7	1.16	1.55	/
DN16711	PiggyBac transposable element-derived protein 4-like	2.85	1.86	/
DN18234	DNA excision repair protein haywire	1.11	/	/
DN19321	DNA helicase	1.56	/	/
DN20562	Collagen alpha-1(II)	2.77	1.56	/

FC, fold change. |log2 FC| > 1 indicates significant difference. “/” indicates no significant differences (negative binomial (NB) distribution, *p* > 0.05): Sf–V, *S. furcifera* females acquired SRBSDV; Sf–NV, *S. furcifera* females failed to acquire SRBSDV; CK, *S. furcifera* females exposed to healthy rice plants.

## Data Availability

Not applicable.

## Supplementary Materials

The following supporting information can be downloaded at: https://www.mdpi.com/article/10.3390/insects13020182/s1, Table S1: Sequences of primers used for RT–qPCR.
