# Supplementary material for: Comparison of Transcriptome Responses between Sogatella furcifera Females That Acquired Southern Rice Black-Streaked Dwarf Virus and Not
Source: Insects. 2022 Feb 9;13(2):182. doi: 10.3390/insects13020182 (PMC8877124; doi:10.3390/insects13020182)
Supplement: Supplementary file 1 [file insects-13-00182-s001.zip › insects-1576568-supplementary.pdf]

Table S1. Sequences of primers used for RT-qPCR of *Sfcas1* and female-specific genes

Primers	Sequences (5'-3')
*Sfcas1*_F	AGTTTCGCTCAGGACCAAGA
*Sfcas1*_R	AGTTGTCGGAATCGGTTGAC
DN16711_F	TGGCCAGTATCAGACATGGA
DN16711_R	CGGATCCTCTGTGAGTGGTT
DN19321_F	GGCGGACGAATCAAGTTCTA
DN19321_R	TTGTTGAGAGACAGCCAACG
DN22835-F	CATTGTTTGATGAGCGGGTA
DN22835-R	AATGGCGTGTTAGCTTGGAC
DN18234-F	GAAACAGCCGATGATGGAGT
DN18234-R	GACGACACACTGGCTCTGAA
DN18646-F	CGAGCCACAAATGTAGACGA
DN18646-R	GCCAGTTCCTTGTCTTGAGC
DN20562-F	TTTCAGCAACCTGCTCTGTG
DN20562-R	ACTGGCTGTTGCTGACACTG
SfEF1α-F	ATTGTGCTGTGCTGATTGT
SfEF1α-R	TGCTCACCTCCTTCTTGAT
